# Current and historic patterns of chronic disease burden are associated with physical activity and sedentary behavior in older adults: an observational study

**DOI:** 10.1186/s12889-025-22264-8

**Published:** 2025-03-17

**Authors:** Mikael Anne Greenwood-Hickman, Rod L. Walker, Abisola E. Idu, John Bellettiere, David Wing, Susan M. McCurry, Paul K. Crane, Eric B. Larson, Dori E. Rosenberg, Andrea Z. LaCroix

**Affiliations:** 1 Kaiser Permanente Washington Health Research Institute, Seattle, WA USA; 2 Herbert Wertheim School of Public Health and Human Longevity Science, University of California San Diego, La Jolla, CA USA; 3 Exercise and Physical Activity Resource Center, Herbert Wertheim School of Public Health and Human Longevity Science, University of California San Diego, La Jolla, CA USA; 4 School of Nursing, University of Washington, Seattle, WA USA; 5 School of Medicine, University of Washington, Seattle, WA USA

**Keywords:** Multimorbidity, Charlson comorbidity index, Accelerometer, Sedentary behavior, Moderate-to-vigorous physical activity (MVPA)

## Abstract

**Background:**

Cross-sectional studies suggest that chronic disease burden in older adults is associated with lower activity. However, preceding life-course patterns of morbidity accumulation may also influence current activity and have not been well characterized. Using a well-described sample of older adults, we estimated associations between current chronic disease burden and accelerometer-measured moderate-to-vigorous intensity movement measures, light-intensity movement measures, and sedentary behavior measures. Additionally, we examined historic morbidity patterns among those with current multimorbidity to provide additional understanding of these later life associations between current multimorbidity and activity.

**Methods:**

Analyses included *N* = 886 older adult study participants who wore activPAL and Actigraph accelerometers. We calculated Charlson Comorbidity Index (CCI; range 0–29) scores for participants at the time of device wear and estimated the association between current chronic disease burden (CCI_current_) and each accelerometer-based activity metric using linear regression. Additionally, for participants categorized as having multimorbidity at time of device wear (CCI_current_ = 2+), we calculated CCI scores from age 55 through age at device wear. We plotted these to illustrate historic patterns of morbidity accumulation, and we compared activity metrics between participants with observed distal vs. recent onset of multimorbidity.

**Results:**

A unit increment in CCI_current_ was associated with higher mean sitting bout duration (0.5 min, CI: [0.0,1.0], *p* = 0.039) and with both lower average daily step counts (-319 steps, CI: [-431,-208], *p* < 0.001) and lower average daily minutes of moderate-to-vigorous physical activity (MVPA; -3.8 min, CI: [-5.2,-2.4], *p* < 0.001). No associations were seen with standing, light-intensity physical activity, or other sitting measures. Among older adults with multimorbidity at time of device-wear, results suggested some evidence that participants whose apparent onset was more distal engaged in less MVPA (-12.1, CI: [-21.0, -3.2], *p* = 0.008) and had fewer daily steps (-1000, CI: [-1745, -254], *p* = 0.009) than participants whose apparent onset was more recent.

**Conclusions:**

Current chronic disease burden was associated with moderate-to-vigorous intensity movement measures and some patterns of prolonged sitting. Current multimorbidity is characterized by a preceding pattern of accumulation over the life-course. Attention to both current and historic trajectory of multimorbidity is important in investigations of MVPA and health.

**Supplementary Information:**

The online version contains supplementary material available at 10.1186/s12889-025-22264-8.

## Background

The prevalence of multimorbidity– the simultaneous presence of two or more chronic conditions in an individual [1]– has climbed steeply in recent years [2–4]. Approximately 50% of adults age 45–64 years and 81% of adults age 65 years and older have two or more chronic conditions [5]. Regular physical activity (PA) and minimizing sedentary behavior (SB) are known to be associated with better health, fewer chronic diseases, and lower mortality risk [6, 7], particularly among individuals with existing multimorbidity [8]. However, multimorbidity can make PA more challenging– especially moderate and vigorous levels of PA– and individuals with chronic conditions in late life, particularly serious conditions such as diabetes [9], chronic obstructive pulmonary disease [10], and cancer [11], are less active than people of similar ages without those conditions. There is also evidence that people with multimorbidity have higher self-reported SB [12, 13]. While the self-report of SB can shed more light on context and type of behavior, it often underestimates SB time and doesn’t allow for the exploration of detailed patterns of SB (e.g., how often SB bouts are interrupted by standing or moving) [14–16], which may be independently associated with health outcomes [17, 18].

Most studies to date examining relationships between multimorbidity and PA and SB consider only current multimorbidity burden and do not characterize or otherwise consider how decades of multimorbidity earlier in the life course might also influence activity in older adults. Furthermore, many studies have documented associations between later-life PA and SB, and mortality and morbidity outcomes, but concerns of reverse causality (i.e., that disease burden in later life may be limiting PA) often limit interpretation [19, 20]. To date, while some studies have explored predictors of disease burden trajectories, including PA [21, 22], and others have examined whether disease burden trajectories predict mortality and other health care utilization outcomes [23–26], we are not aware of any studies that have examined both current and historical multimorbidity in association with current PA and SB in later life.

Characterizing morbidity over time can be challenging as it requires extensive and detailed historical data that in many instances are not available to researchers. Summary index measures that use administrative claims data to capture certain severe chronic conditions in an aggregate score can offer a pragmatic approach to characterizing multimorbidity [27]. Here, we use device-based assessments of PA and SB in a cohort of older adults and leverage the Charlson Comorbidity Index [27, 28] which has been documented to be predictive of healthcare utilization and overall quality of life [29], to: (1) assess whether current chronic disease burden was associated with current PA and SB, and (2) examine patterns of historical morbidity accumulation among participants with current multimorbidity to provide additional understanding of the complex relationship between chronic disease burden and PA and SB during late life.

## Methods

### ACT activity monitoring cohort

The Adult Changes in Thought (ACT) Activity Monitoring sub-study was described in detail previously [30]. Briefly, the ACT Study is an on-going longitudinal cohort study of older adults (age 65 + years) that began in 1994 to investigate risk factors for dementia. Dementia-free participants are randomly sampled from the membership panels of Kaiser Permanente Washington (KPWA) clinics in King County, WA. KPWA (previously Group Health) is an integrated healthcare delivery system providing care in Washington State and Idaho. ACT participants undergo biennial assessments for the purpose of tracking cognition and the onset of dementia [31]. Starting in 2016, ACT added device-based activity monitoring (ACT Activity Monitor Sub-Study, ACT-AM). If participants were a wheelchair user, receiving hospice or care for a critical illness, residing in a nursing home, or if memory problems became evident during their most recent study visit, they were not eligible to participate in ACT-AM. Those choosing to participate provided written informed consent. All procedures were approved by the KPWA institutional review board.

### Physical activity and sedentary behavior measurement

Two devices were used to provide measures of SB and PA: the activPAL micro3 (PAL Technologies, Glasgow, Scotland, UK) and the ActiGraph wGT3X+ (ActiGraph LLC, Pensacola, FL, USA). The activPAL was affixed with a waterproof dressing to the front center thigh and the ActiGraph was worn on an elastic belt situated so the device rested at the right suprailiac crest. A 24-hour wear protocol was used for both devices for one week. In-bed time was captured through a daily sleep log, and this self-reported in-bed time was removed from analysis to limit capture to waking hours. To be included, a minimum of 4 days with 10 or more hours of waking wear time was required for each device of interest [32].

Event-level files were extracted using proprietary PAL Technologies software and sleep time was removed using a batch processing program in R. The activPAL is more accurate for measuring SB compared to hip-based accelerometry [33] and was therefore used exclusively for SB outcomes. Daily summary activPAL-derived measures included: mean total sitting time (mins/day), mean total standing time (mins/day), mean total sit-to-stand transitions (number/day), mean sitting bout duration (mins/bout), mean number of sitting bouts ≥ 30 min (number/day), and mean total steps.

Raw ActiGraph data were collected at 30 Hz and processed into a proprietary counts variable at 15-second epochs using the normal filter in the ActiLife software (v 6.13.3). Cutpoints calibrated for older adults developed in a Women’s Health Initiative laboratory study were applied to the data [34]. Data were then summarized at the participant-level across all adherent days to generate daily behavior summaries. Specifically, intensity classifications using vector magnitude counts per 15-second epoch were as follows: ≤18 for SB, 19–518 for light-intensity physical activity (LPA), and > 518 for moderate-to-vigorous physical activity (MVPA). Daily summary Actigraph measures included: mean total time in LPA (mins/day) and mean total time in MVPA (mins/day).

### Chronic disease burden

Chronic disease burden was measured using the Charlson Comorbidity Index (CCI; range 0–29, higher scores suggest higher morbidity burden) [27, 28], which accounts for history of myocardial infarction, congestive heart disease, peripheral vascular disease, cerebrovascular disease, dementia, chronic pulmonary disease, rheumatologic diseases, peptic ulcer disease, liver disease, diabetes, hemiplegia or paraplegia, renal disease, solid tumors, malignancies including leukemia and lymphoma, and acquired immunodeficiency syndrome (AIDS). Supplemental File 1 details the ICD-9/10 diagnosis codes used to compute CCI.

Current CCI score (CCI_current_, i.e., current chronic disease burden) was calculated based on ICD-9/10 diagnosis codes present in the participant’s electronic medical record over the year prior to device wear, using the date of the ACT assessment visit at which devices were distributed as the index date. Participants who were not continuously enrolled in the health system over that prior year had missing values for CCI_current_ unless they were continuously enrolled over the one-to-two-year period prior to the index date, in which case we imputed CCI_current_ using diagnosis codes from that proximal time window.

To calculate morbidity over time, individuals’ birthdays were used as the index date annually to calculate their CCI score starting at age 55 or their earliest year of enrollment in KPWA post-1993 (when electronic records of diagnosis codes are accessible) if after age 55. We used diagnosis codes from any inpatient, outpatient, emergency department, non-acute institutional stay (e.g., skilled nursing facility, hospice, etc.), or other encounter within the KPWA system in the previous year to calculate a CCI score for that age. We repeated this process annually through each participant’s age at time of device wear during the 2016–2018 ACT biennial cycle. If a participant was not enrolled in KPWA in a given year, CCI was missing for that age.

### Covariates

Covariates were collected from the ACT study visit most proximal to the date of device wear (typically the first day of device wear). Age (years; continuous), gender, race/ethnicity, education (some college/post-secondary vs. high school or less), marital status, depressive symptoms from the Center for Epidemiologic Studies Depression Scale (CES-D; scores ≥ 10 indicate significant depressive symptoms vs. scores < 10) [35, 36], smoking status (current/prior vs. never), and history of any alcohol problem were assessed via participant interview. History of any alcohol problem was defined as a participant reporting ever having had: a doctor suggest to cut down on drinking; a social, marital, or work-related problem due to drinking; aggressive behavior while under the influence of alcohol; or, 2 or more traffic violations associated with alcohol. Body mass index (BMI; kg/m^2^; continuous) was calculated from height and weight measured by study staff during the visit.

At the time of this study, male or female were the only reportable gender options. Given that the study did not ask about sex assigned at birth, it is possible the study variable may reflect gender or sex. Race/ethnicity categories were obtained via self-report and do not represent biological or genetic meaning, but rather represent participant self-identified racial and ethnic identities in a healthcare research setting and serve as a proxy indicator for potential inequities in health, healthcare, and behavioral impacts driven by structural and systemic racism [37, 38]. The sample was characterized using all reportable categories available at the time of this study (Hispanic ethnicity yes/no and, separately, American Indian/Alaska Native, Asian, Black, Native Hawaiian or Other Pacific Islander, White, Other Race, More than One Race, and Unknown/Not Reported); however, due to small sample sizes in many groups, categories were consolidated (non-Hispanic white vs. other) in regression models.

### Statistical analysis

For each PA and SB outcome, we fit a linear regression model to estimate the cross-sectional association between CCI at time of device wear (CCI_current_) and the outcome. All outcome models were adjusted for age, sex, race/ethnicity, education, BMI, depressive symptoms, and device wear time. We additionally investigated potential effect modification by sex and age through use of interaction terms. Primary analyses included CCI_current_ as a continuous variable (assumed linear relationship with the outcome). In sensitivity analyses, we estimated associations categorizing CCI_current_ as 0, 1, 2, 3 + and also explored the possibility of a non-linear relationship through use of loess curves and cubic polynomials. In additional sensitivity analyses, we repeated analyses with additional model adjustment for smoking, alcohol, and marital status. Estimates were provided with 95% confidence intervals (CI), and statistical significance was assessed at the *p* < 0.05 level.

To account for potential selection bias related to device-wear and to yield results generalizable to the broader ACT cohort, we applied inverse probability weighting for accelerometer outcome models. In alignment with prior analyses for this cohort [30, 39], we used the broader ACT sample initially eligible for participation in ACT-AM to estimate a logistic regression model for the binary outcome of device-wear consent with returned valid data for both activPAL and ActiGraph as a function of several key demographic (age in years; gender; non-Hispanic white vs. all other racial/ethnic categories; education at least post-secondary vs. less), ACT assessment location (in clinic vs. home), and health-related (BMI in kg/m^2^; CES-D 10 + vs. <10; self-rated health on 5-point scale from 1 = excellent through 5 = poor; difficulty with self-reported activities of daily living as a count from 0 to 6) covariates. Predictions from this model were used to construct inverse probability of response (consent + returned valid data) weights which were then incorporated in all outcome models [40]. Standard errors were estimated via the sandwich estimator.

For participants with current multimorbidity (CCI_current_ = 2+), we further provided graphical depictions of historic life-course patterns of morbidity accumulation (CCI score) prior to device wear. Given that the extent of historical CCI information available from the health system depends on both a participant’s current age, as well as their duration enrolled in the health system, these plots were stratified by age at time of device wear in 5-year grouping (i.e., 65–69, 70–74, 75–79, 80–84, 85–89, and 90+) and restricted to participants with at least 10 years of historical data available (96% of the 2 + group). For each plot a smoothed loess curve was provided to depict the average CCI over time.

As a secondary analysis, we then dichotomized these current multimorbidity participants into two groups: those who had an observed CCI of 2+ (i.e., multimorbidity) 8 or more years prior to device wear vs. those who did not. For ease, the former group we refer to as ‘distal multimorbidity onset’ and the latter we refer to as ‘recent multimorbidity onset’. We chose to dichotomize at 8 years for this investigation as 8 was the median of the distribution of years since earliest observed multimorbidity. We then estimated the difference in means of the PA and SB outcome activities between the distal and recent onset groups using linear regression models adjusted for CCI_current_ and the same set of adjustment covariates as in primary analyses. We only performed this analysis for outcomes that had shown significant associations with CCI_current_ in primary analyses.

Data processing and modeling used R, version 4.3.2 (R Foundation for Statistical Computing, Vienna, Austria), and SAS, version 9.4 (SAS Institute, Inc., Cary, NC).

## Results

### Sample demographics

Overall, 1,885 ACT participants met eligibility criteria and were approached to participate in the ACT-AM study. Of these, 951 ultimately consented to wear, and had 4 + valid days of data on, both devices. Consenting individuals were younger and healthier than non-consenters [30]. Of the 951 with valid wear, 30 (3%) were excluded from the analytic sample due to some missing covariate information to be used in primary analyses and 35 were excluded (4%) due to missing data on CCI_current_, leaving a final analytic sample of *N* = 886 (Supplemental File 2).

Table 1 displays demographic and health characteristics for the final sample overall and by levels of CCI_current_. The overall sample had a mean age of 77 at time of activity monitoring, was 56% women, 90% non-Hispanic white, 3% Asian, 1% Black, 1% Hispanic (regardless of racial identity), and 5% self-identifying as another racial group. Relative to the CCI_current_ = 0 and 1 groups, the CCI_current_ = 2+ group included smaller proportions of women (46% vs. 59% and 57%), non-Hispanic white participants (84% vs. 92% and 92%), and participants with at least some post-secondary education (88% vs. 92% and 93%). The proportion of participants reporting significant depressive symptoms was also higher in the CCI_current_ = 2+ group (14% vs. 7% and 5% in the CCI_current_ = 0 and 1 groups, respectively).

**Table 1:** Characteristics of included sample at time of activity monitoring by current chronic disease burden (CCI_current_)

	Overall *N* = 886	CCI_current_ = 0 *N* = 515	CCI_current_ = 1 *N* = 137	CCI_current_ = 2+ *N* = 234
Age, mean (SD)	77.0 (6.9)	75.5 (6.3)	76.7 (6.8)	80.3 (7.1)
Women, n (%)	492 (55.5)	306 (59.4)	78 (56.9)	108 (46.2)
Hispanic Ethnicity, n (%)	12 (1.4)	7 (1.4)	2 (1.5)	3 (1.3)
Race, n (%)				
Asian	25 (2.8)	13 (2.5)	2 (1.5)	10 (4.3)
Black	12 (1.4)	3 (0.6)	1 (0.7)	8 (3.4)
Native Hawaiian / Pacific Islander	2 (0.2)	0 (0.0)	1 (0.7)	1 (0.4)
White	807 (91.1)	481 (93.4)	127 (92.7)	199 (85.0)
Other Race / More than One Race / Not Reported	40 (4.5)	18 (3.5)	6 (4.4)	16 (6.8)
Non-Hispanic White^a^, n (%)	796 (89.8)	474 (92.0)	126 (92.0)	196 (83.8)
Post-Secondary Education, n (%)	807 (91.1)	474 (92.0)	127 (92.7)	206 (88.0)
BMI, mean (SD)	27.0 (5.0)	26.3 (4.6)	28.0 (5.8)	27.7 (5.2)
Depressive Symptoms^b^, n (%)	76 (8.6)	37 (7.2)	7 (5.1)	32 (13.7)
Current / Prior Smoking^c^, n (%)	398 (45.1)	211 (41.0)	65 (47.8)	122 (52.4)
Ever Alcohol Problem^d^, n (%)	167 (18.9)	95 (18.5)	20 (14.6)	52 (22.3)
Marital Status, n (%)				
Never Married	53 (6.0)	38 (7.4)	7 (5.1)	8 (3.4)
Married / Living as Married	516 (58.2)	310 (60.2)	87 (63.5)	119 (50.9)
Separated / Divorced	160 (18.1)	88 (17.1)	22 (16.1)	50 (21.4)
Widowed	148 (16.7)	74 (14.4)	18 (13.1)	56 (23.9)
Other	9 (1.0)	5 (1.0)	3 (2.2)	1 (0.4)
Current CCI, mean (SD)	1.0 (1.6)	0.0 (0.0)	1.0 (0.0)	3.2 (1.6)

^a^ A combined race/ethnicity variable was used in modeling

^b^ Depressive symptoms defined as a score of 10 or greater on the Center for Epidemiologic Studies Depression Scale (CES-D)

^c^ Of the 398 participants reporting current or prior smoking, only 14 reported current. 3 participants were missing information, so reported n (%) is among the non-missing

^d^ Ever alcohol problem defined as participant reporting ever having had: a doctor suggest to cut down on drinking; a social, marital, or work-related problem due to drinking; aggressive behavior while under the influence of alcohol; or, 2 or more traffic violations associated with alcohol. 2 participants were missing information, so reported n (%) is among the non-missing

### Primary outcome modeling

Table 2 displays descriptive summaries of SB and PA outcomes of interest by level of CCI_current_ along with estimated coefficients from all outcome models by activity type (sedentary, light-intensity movement, and moderate-to-vigorous intensity movement), adjusted for age, sex, race/ethnicity, education, BMI, depressive symptoms, and device wear time.

**Table 2:** Sedentary behavior and physical activity summaries and estimated associations with current chronic disease burden (CCI_current_)

		Overall *N* = 886	CCI_current_ = 0 *N* = 515	CCI_current_ = 1 *N* = 137	CCI_current_ = 2+ *N* = 234	Cross-Sectional Modeling Results for CCI_current_ β (95% CI)^d^
Sedentary Behavior Measures	**Daily Total Sitting (minutes)**^**a**^, **mean (SD)**	599 (116)	583 (113)	598 (110)	633 (117)	5.4 (-0.2, 11.1)
	**Mean Bout Duration (minutes)**^**a**^, **mean (SD)**	15.6 (7.3)	14.7 (6.4)	15.0 (5.2)	18.0 (9.3)	0.5 (0.03, 1.0)*
	**Sitting bouts > 30 min (n/day)**^**a**^, **mean (SD)**	5.8 (1.7)	5.6 (1.7)	5.9 (1.7)	6.2 (1.7)	0.0 (-0.03, 0.1)
	**Sit-to-stand transitions (n/day)**^**a**^, **mean (SD)**	43.7 (12.8)	44.8 (12.8)	43.9 (11.9)	41.3 (12.9)	-0.5 (-1.1, 0.1)
Light-Intensity Movement Measures	**Standing Time (minutes)**^**a**^, **mean (SD)**	243 (95)	253 (91)	237 (88)	225 (104)	-1.9 (-6.9, 3.2)
	**LPA (minutes)**^**b, c**^, **mean (SD)**	277 (76)	288 (75)	267 (66)	258 (77)	-1.7 (-5.1, 1.7)
Moderate-to-Vigorous Intensity Movement Measures	**Steps**^**a**^, **mean (SD)**	6895 (3505)	7650 (3586)	6636 (2885)	5385 (3137)	-319 (-431, -208)*
	**MVPA (minutes)**^**b, c**^, **mean (SD)**	69 (44)	78 (44)	68 (39)	49 (39)	-3.8 (-5.2, -2.4)*

^a^ activPAL measures: daily total sitting time, mean sitting bout duration, number daily sitting bouts > 30 min, number daily sit-to-stand transitions, daily total standing time, daily total steps

^b^ ActiGraph measures: daily total LPA, daily total MVPA

^c^ LPA and MVPA defined using Objective Physical Activity and Cardiovascular Health in older Women (OPACH) cutpoints, which are validated for an older adult population

^d^ The β parameter (with 95% CI) corresponds to the estimated change in the mean of the given sedentary behavior or physical activity measure associated with a 1-unit increase in CCI_current_; model adjusted for age, sex, race/ethnicity, education, BMI, depressive symptoms, and device wear time

Notes: CCI = Charlson Comorbidity Index; CI = Confidence Interval; PA = Physical Activity; LPA = Light-Intensity Physical Activity; MVPA = Moderate-to-Vigorous Physical Activity

*Statistically significant associations at the *p* < 0.05 level

### Sedentary behavior measures

Of all sedentary behavior metrics, CCI_current_ was only associated with mean sitting bout duration. A 1 point higher CCI_current_ was associated with a mean sitting bout duration approximately 0.5 min longer (0.5, CI: [0.0,1.0], *p* = 0.039).

### Light-intensity movement measures

Neither total daily standing time nor total daily time in LPA were associated with CCI_current_.

### Moderate-to-Vigorous intensity movement measures

Each additional point on the CCI_current_ score was associated with 319 fewer daily steps (-319, CI: [-431,-208], *p* < 0.001) and approximately 4 fewer minutes in daily MVPA (-3.8, CI: [-5.2,-2.4], *p* < 0.001).

### Interaction analyses

Investigations of effect modification did not find evidence (*p* < 0.05) of differences in any of the CCI_current_-outcome associations by sex but did by age (though magnitudes were small). Specifically, for the moderate-to-vigorous intensity movement measures of steps and MVPA, associations with CCI_current_ were attenuated with older age such that each year increase in age attenuated the CCI_current_-steps association by 15 daily steps (15, CI: [2, 28], *p* = 0.020) and the CCI_current_-MVPA association by 0.2 min of daily MVPA (0.2, CI: [0.1, 0.4], *p* = 0.001).

### Sensitivity analyses

Results from sensitivity analyses using a categorical representation of CCI_current_ as 0, 1, 2, and 3 + are provided in Table 3. Conclusions regarding associations are similar to those drawn from primary analyses, though in some instances, estimated differences were of greater magnitude than suggested by primary analyses (e.g., when comparing between the 0, 1, and 2 groups, specifically). Explorations of non-linearity in the relationship between CCI_current_ and each outcome measure did not lead to changes in conclusions. Graphical comparisons of linear vs. loess or cubic polynomial based models suggested little difference in the relationships across most of the CCI_current_ values and only tended to deviate, if at all, toward the extremes (e.g., CCI_current_ = 7+) where data were sparse (data not shown). Sensitivity analyses that included additional model adjustment for smoking, alcohol, and marital status did not yield meaningfully different results from primary analyses without such adjustment (Supplemental Files 3–4).

**Table 3:** Sensitivity analyses^e^ of sedentary behavior and physical activity associations with current chronic disease burden (CCI_current_)

		CCI_current_ = 0 *N* = 515	CCI_current_ = 1 *N* = 137 β_1vs.0_ (95% CI)	CCI_current_ = 2 *N* = 114 β_2vs.0_ (95% CI)	CCI_current_ = 3+ *N* = 120 β_3+vs.0_ (95% CI)	*P*-value^d^
Sedentary Behavior Measures	**Daily Total Sitting (minutes)** ^**a**^	Reference	6.1 (-16.2, 28.4)	18.6 (-7.8, 45.0)	36.5 (10.7, 62.2)	0.042*
	**Mean Bout Duration (minutes)** ^**a**^	Reference	-0.7 (-1.8, 0.4)	2.3 (0.3, 4.3)	2.3 (0.2, 4.4)	0.003*
	**Sitting bouts > 30 min (n/day)** ^**a**^	Reference	0.0 (-0.3, 0.4)	0.1 (-0.3, 0.4)	0.3 (0.0, 0.6)	0.342
	**Sit-to-stand transitions (n/day)** ^**a**^	Reference	0.9 (-1.4, 3.2)	-1.6 (-4.4, 1.2)	-2.3 (-5.1, 0.5)	0.167
Light-Intensity Movement Measures	**Standing Time (minutes)** ^**a**^	Reference	-1.3 (-21.1, 18.5)	-9.9 (-34.0, 14.1)	-19.1 (-41.9, 3.6)	0.389
	**LPA (minutes)** ^**b, c**^	Reference	-7.8 (-19.9, 4.3)	-16.6 (-30.9, -2.3)	-12.2 (-27.2, 2.7)	0.087
Moderate-to-Vigorous Intensity Movement Measures	**Steps** ^**a**^	Reference	-417 (-907, 74)	-848 (-1450, -246)	-1516 (-2085, -946)	< 0.001*
	**MVPA (minutes)** ^**b, c**^	Reference	-2.9 (-9.4, 3.5)	-9.0 (-16.1, -1.9)	-19.2 (-25.8, -12.6)	< 0.001*

^a^ activPAL measures: daily total sitting time, mean sitting bout duration, number daily sitting bouts > 30 min, number daily sit-to-stand transitions, daily total standing time, daily total steps

^b^ ActiGraph measures: daily total LPA, daily total MVPA

^c^ LPA and MVPA defined using Objective Physical Activity and Cardiovascular Health in older Women (OPACH) cutpoints, which are validated for an older adult population

^d^ P-value corresponds to a joint (omnibus) test of the 3 parameters corresponding to the exposure contrasts (β_1vs.0_, β_2vs.0_, β_3+vs.0_)

^e^ Sensitivity analyses in which CCI_current_ is modeled as a categorical variable (0, 1, 2, 3+) rather than as a continuous term; model adjusted for age, sex, race/ethnicity, education, BMI, depressive symptoms, and device wear time

Notes: CCI = Charlson Comorbidity Index; CI = Confidence Interval; PA = Physical Activity; LPA = Light-Intensity Physical Activity; MVPA = Moderate-to-Vigorous Physical Activity

*Statistically significant associations at the *p* < 0.05 level

### Multimorbidity over age

**Fig. 1:**
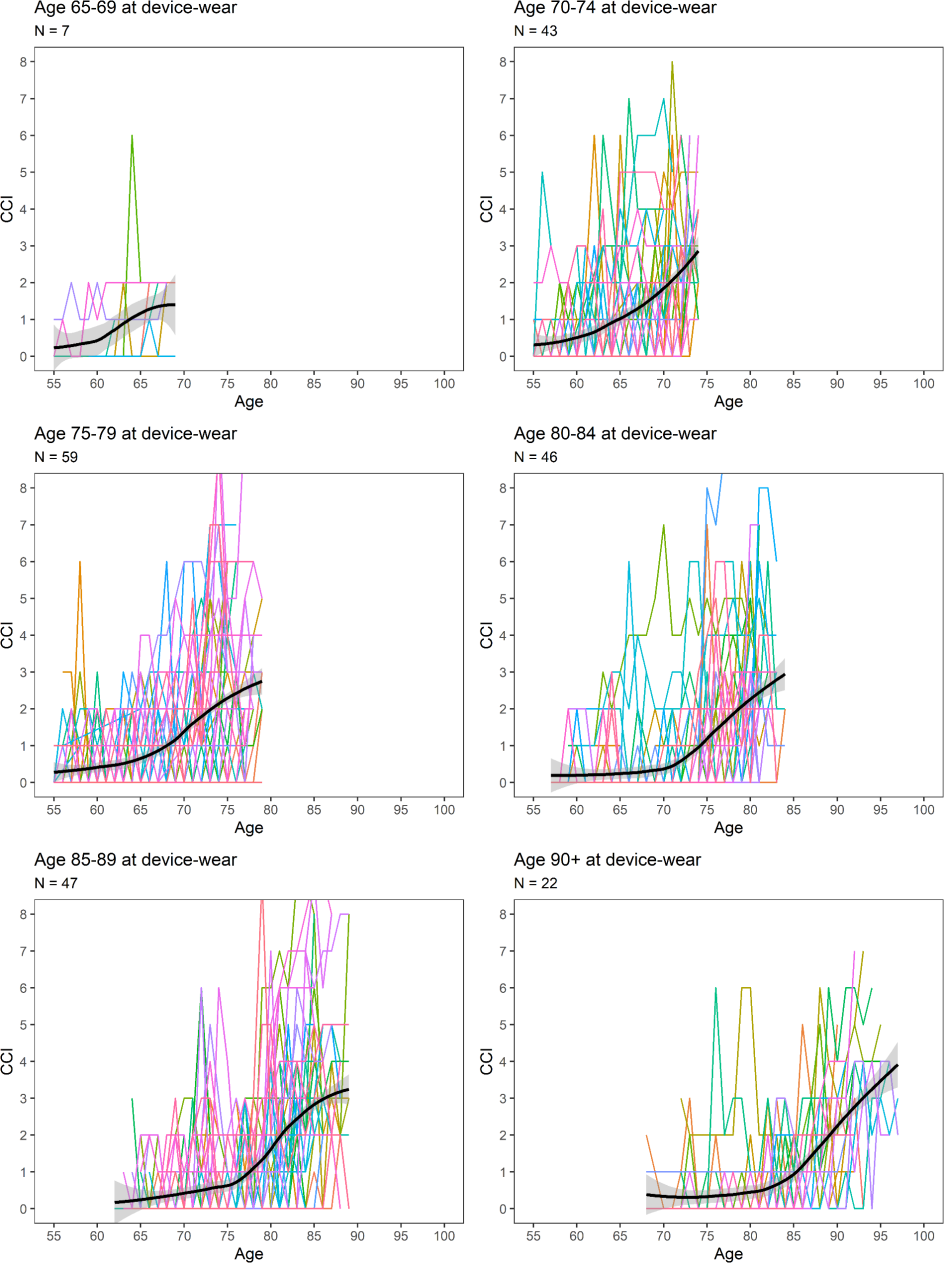
Life-course patterns of morbidity accumulation over age. Data shown is among participants with current chronic disease multimorbidity (CCI_current_ = 2+) and at least 10 years of historical data. Colored lines represent individual participant trajectories of Charlson Comorbidity Index (CCI) over age. Solid black lines represent smoothed loess curves to depict the average CCI over age

Figure 1 depicts historic life-course patterns of morbidity accumulation prior to device wear for participants with current multimorbidity (CCI_current_ = 2+) and sufficient prior enrollment (10 + years). A feature first notable from these plots is that there is a high degree of variability in individual trajectories preceding participants’ current assessed multimorbidity. Given the construct of how CCI is computed (based on diagnosis codes in distinct 1-year periods over time), this likely reflects a mix of increasing development of chronic conditions combined with variability in individual healthcare utilization over time. While individual trajectories varied widely, comparing the average trend observed in these plots, we also observe that the older the age at device wear, the older the age at multiple morbidity accumulation, which may, at least in part, be a reflection of age and health selection factors influencing who was willing and able to be included in the physical activity measurement sub-study.

Table 4 provides results from our closer investigation of this current multimorbidity sample, comparing mean sitting bout duration, daily steps, and daily MVPA between participants with distal vs. recent multimorbidity onset. Results suggested some evidence that participants whose apparent onset was more distal currently engaged in less MVPA (-12.1, CI: [-21.0, -3.2], *p* = 0.008) and had fewer daily steps (-1000, CI: [-1745, -254], *p* = 0.009) than participants whose apparent onset was more recent. Results from sensitivity analyses with additional model adjustment for smoking, alcohol, and marital status were similar (Supplemental File 5).

**Table 4:** Comparison of sedentary behavior and physical activity between participants^e^ with distal vs. recent multimorbidity onset

	Recent onset (< 8 years) *N* = 105	Distal onset (≥ 8 years) *N* = 119	Adjusted difference in means Β_Distal−Recent_ (95% CI)^d^
Mean Bout Duration (min)^a^, mean (SD)	16.6 (6.3)	19.3 (11.0)	2.5 (-0.03, 5.0)
Steps^a^, mean (SD)	6025 (3351)	4603 (2506)	-1000 (-1745, -254)*
MVPA (min)^b, c^, mean (SD)	58 (41)	39 (32)	-12.1 (-21.0, -3.2)*

^a^ activPAL measures: mean sitting bout duration, daily total steps

^b^ ActiGraph measure: daily total MVPA

^c^ MVPA defined using Objective Physical Activity and Cardiovascular Health in older Women (OPACH) cutpoints, which are validated for an older adult population

^d^ The β_Distal−Recent_ parameter (with 95% CI) corresponds to the estimated model-adjusted difference in means of the given activity measure between participants with distal multimorbidity onset vs. recent multimorbidity onset; model adjusted for age, sex, race/ethnicity, education, BMI, depressive symptoms, and device wear time

^e^ Among participants with current chronic disease multimorbidity (CCI_current_ = 2+) and at least 10 years of historical data

Notes: CI = Confidence Interval; PA = Physical Activity; MVPA = Moderate-to-Vigorous Physical Activity

*Statistically significant associations at the *p* < 0.05 level

## Discussion

In a well-characterized sample of older men and women, we used electronic health record data and device-measured summaries of PA and SB to estimate associations between current chronic disease burden and daily activity at various intensities. Our results indicated that higher chronic diseased burden as measured by CCI_current_ was significantly associated with patterns of more prolonged and uninterrupted sitting (i.e., slightly greater mean sitting bout duration) and with less PA, particularly at higher intensities (steps and MVPA) in later life.

In the older adult sample studied here, current chronic disease burden was associated with one of the considered sedentary patterns (i.e., mean sitting bout duration). The magnitude of this association was small, however, around 0.5 min/bout for each unit increase in CCI_current_, representing a change of about 3% from the sample average. When compared to evidence from sedentary behavior interventions where a change of 1.8 min in mean sitting bout duration was noted along with clinically significant reductions to blood pressure [41, 42], it is unclear if a 30-second difference in this metric is indicative of a meaningful difference in the average sitting bout length. Future intervention studies would be needed to determine how much older adults with multimorbidity can change the average length of their sitting bouts and examine whether such associations meaningfully improve health. In this sample we do note a higher prevalence of depressive symptoms in the multimorbidity (CCI_current_ = 2+) group that is twice that of the other CCI groups. Multimorbidity and physical limitations, as well as lack of self-efficacy and fear, have been reported previously as major barriers to many older adults in pursuing PA as well as directly facilitating their sedentary habits [43–45]. Late-life SB is likely not just driven by chronic disease burden but also by social and health-related factors beyond those measured here, such as social sitting norms for older adults or chronic pain [46, 47]. Furthermore, there is growing evidence that different sedentary activities can have differential relationships with health outcomes. For instance, socializing or engaging in other mentally active sedentary behaviors may have positive health impacts, while more passive activities like watching television, may have deleterious health effects [48–50]. Exploration of a more complete pathway, including analysis of the types of sedentary activities people engage in, may be helpful in future studies to fully elucidate the relationship between sitting time and current chronic disease burden.

We did not note any significant associations between current chronic disease burden and LPA or standing time. This may reflect the types of activities that compose most daily standing and light movement. Specifically, LPA, defined in these analyses as movement between 1.5 and 3.0 metabolic equivalents (METs) [34], comprises activities such as walking or moving slowly, lifting light objects, and light housework or gardening [51, 52]. Taken together, standing and LPA time generally represent the vast majority of an individual’s time spent completing activities of daily living (ADLs) and instrumental ADLs (I-ADLs), such as meal preparation, personal hygiene, and light household chores [51, 53]. These activities, typically performed as part of daily independent living and not related to recreational exercise, are less discretionary, do not require moderate or vigorous exertion, and thus, may be more immune to the influence of chronic disease burden, at least as characterized by CCI. In older adults with higher chronic disease burdens and declining physical and cognitive abilities, it is well known that ADL and I-ADL performance eventually can become impaired [53, 54]. However, the ACT-AM sample is healthier than the broader ACT cohort [30], so our analyses may have been limited by lack of individuals across the full range of impairment (CCI_current_ was 0 or 1 for 74% of our sample). These findings could support the need for SB reduction interventions that encourage breaking up sitting time, which could be done by increasing standing, LPA, or MVPA. Given that older populations with multimorbidity have barriers to MVPA, an approach focusing on reducing prolonged sitting via more standing or movement at light intensities might be more practical as evidenced by some recent trials [41, 55–57].

For MVPA and steps, there were associations with current chronic disease burden, though the magnitudes were modest, with an average decrease of about 300 steps and 4 min of daily MVPA associated with each additional point on CCI_current_. Evidence of effect modification by age in these associations, however, suggests they are attenuated at older ages, possibly due to the natural decline in physical activity associated with the aging process itself [58]. Our findings that those with current multimorbidity (CCI_current_ = 2+) who had earlier apparent multimorbidity onset engaged in lower levels of these activities than those with more recent onset may suggest that both current multimorbidity burden, which may directly correlate with physical limitations on activity [59], and historical trajectory of multimorbidity are important predictors of these more intense activity measures in late life. One interpretation of this finding is that earlier multimorbidity (i.e., in mid-life) could make it difficult for people to engage in higher intensity movement because of the many documented physical and mental manifestations of prolonged multimorbidity (e.g., chronic pain, neuropathy, fatigue, generalized weakness, depression) [60, 61]. Further, these symptoms can reduce self-efficacy for exercise, one of the strongest predictors of MVPA [45]. Future studies that directly explore the associations between historic trajectories of multimorbidity and MVPA via these candidate mechanistic pathways are needed to fully elucidate this relationship.

It is clear from these findings that late-life MVPA is often an indicator for both current multimorbidity and historic trajectory of multimorbidity. In other words, associations of MVPA with various health outcomes may be confounded by both historic and current multimorbidity burden. Understanding the complex relationship between MVPA and health likely requires taking a broader life course perspective to better understand what precedes any single point-in-time measurement of activity. When rich historic data are available, leveraging it to directly control for historic health status may be warranted. However, such historic data are rarely available, meaning that at a minimum, adjustment for, and stratification by, multimorbidity in studies of MVPA and health outcomes is critically important for appropriate interpretation. Whether MVPA partially mediates associations between multimorbidity and health outcomes of interest should be investigated, and associations between MVPA and health outcomes, unless adjusted for multimorbidity, are likely confounded by uncontrolled disease burden and the historic trajectory of its accumulation. Furthermore, intervention studies may prove the ideal approach to resolve these questions of reverse causation and inform programs of health promotion. For instance, it is possible that interventions to increase self-efficacy for exercise, manage pain, and mitigate fatigue stemming from chronic conditions over the life-course, particularly those employing a stepped approach starting first with decreasing SB and increasing LPA and ultimately building to increasing levels of MVPA, might increase overall PA and health trajectories in later life. Such a “staircase approach” to decreasing SB and increasing PA has been previously proposed in the context of promoting cardiovascular health [62]. Future studies explicitly exploring such mediators and testing interventions that target them are warranted.

### Strengths & limitations

This study has several strengths. First, the ACT cohort is community-based and drawn from KPWA members, allowing us to leverage medical records data on a large sample to assess multimorbidity (both at the time of device-wear and over time). Due to the nature of Kaiser Permanente as an integrated delivery system, participants would have received nearly all their healthcare within the system when enrolled, so we believe we have consistent capture of multimorbidity for our sample. Further, we use objective measures of PA and SB through participants’ concurrent wear of both the activPAL and ActiGraph. We also employed the CCI to represent multimorbidity burden. The CCI is commonly available in many healthcare settings, is easily leveraged on large populations at multiple time points, and is widely documented in the literature, which increases ease of interpretation and comparison with other studies [29].

However, we also acknowledge several limitations. Importantly, generalizability of these findings is likely limited by the fact that the ACT-AM sample is predominantly non-Hispanic white, and well-educated. Additionally, ACT participants who agreed to participate in ACT-AM were healthier on average than those who did not [30]. Though these analyses countered selection bias of inclusion in the activity monitoring cohort from the larger ACT cohort using inverse probability weighting, it is likely some bias remains, and findings may not be fully generalizable to broader older adult populations. As noted in our methods describing ActiGraph data processing and consistent with previous ACT-AM analyses [30], we employ cut points calibrated specifically for older adult women. While this accounts for age-related increases in the energy requirements of movement and reduces misclassification of MVPA as LPA, the cutpoints have not been validated in men. To our knowledge based on prior comparisons [30], these are the best available cutpoints for older adults, but we acknowledge that some error may exist, given that we have applied them in a sample that includes men. Relatedly, these analyses explore relationships with different activity behaviors independently, but there is growing recognition in the field that compositional data approaches that acknowledge the interrelatedness of the spectrum of movement behaviors across the day are needed [63]. Future work to explore relationships between multimorbidity and different distributions of these behaviors across the 24-hour day is warranted. Our chosen chronic disease burden scale, the CCI, was originally designed as a mortality risk assessment tool. Although it includes a list of serious, life limiting chronic conditions, it does not include some common age-related conditions that may also impact PA and SB such as osteoarthritis, osteoporosis, depression, and others, nor is it able to assess how the interplay of specific conditions may compound to impair function in a way that other combinations do not. No multimorbidity measure is comprehensive in capturing all possible conditions and their severity, and we acknowledge CCI could mis-estimate chronic disease burden for some participants. Additionally, we could not calculate the CCI prior to 1993 as diagnosis codes were not electronically available prior to then. This limited our ability to examine the full CCI trajectory to age 55 for the oldest participants in the sample.

## Conclusions

In sum, these findings suggest that historic patterns and current levels of multimorbidity throughout an individual’s life-course are important determinants of movement behaviors like steps and MVPA and thus should be considered in interpretation of associations between PA and health in later life. More than just a measure of activity, MVPA, particularly for older adults, is likely an overall indicator of health and fitness, and, if it is not adequately adjusted for underlying health status, residual confounding is likely. Furthermore, intervention earlier in the life course to improve PA and SB patterns may be key to improving both activity and health in late life. Future studies to better elucidate the mediators between mid-life disease burden and late life activity behavior, and where in that pathway to intervene most effectively, are warranted.

## Electronic supplementary material

Below is the link to the electronic supplementary material.

Supplementary Material 1

Supplementary Material 2

Supplementary Material 3

Supplementary Material 4

Supplementary Material 5

## Data Availability

Data from this analysis cannot be made publicly available for ethical and legal reasons. In order to replicate our findings, a researcher may need access to personal health identifiers (PHI) including dates of birth and death, dates of diagnoses, and ages over 89. These are required variables for the analysis, and we cannot publicly release this information without IRB approval and a Data Use Agreement with interested researchers. However, the datasets used and/or analyzed in the current study are available upon reasonable request and execution of appropriate human subjects review and data sharing agreements by following the process described on the Adult Changes in Thought (ACT) website: https://actagingstudy.org.

## Abbreviations

CCI: Charlson Comorbidity Index
MVPA: Moderate-to-vigorous physical activity
PA: Physical activity
SB: Sedentary behavior
ACT: Adult Changes in Thought study
ACT-AM: Adult Changes in Thought Activity Monitor sub-study
KPWA: Kaiser Permanente Washington
LPA: Light-intensity physical activity
CES-D: Center for Epidemiologic Studies Depression Scale
BMI: Body mass index
CI: Confidence interval
METs: Metabolic equivalents
ADL: Activities of daily living
I-ADL: Instrumental activities of daily living
