# Supplementary material for: Current and historic patterns of chronic disease burden are associated with physical activity and sedentary behavior in older adults: an observational study
Source: BMC Public Health. 2025 Mar 17;25:1032. doi: 10.1186/s12889-025-22264-8 (PMC11917095; doi:10.1186/s12889-025-22264-8)
Supplement: Supplementary file 1 — Supplementary Material 1 [file 12889_2025_22264_MOESM1_ESM.docx]

**Supplemental File 1**. ICD-9 and ICD-10 codes used to calculate the Charlson Comorbidity Index

| **Diagnosis** | **ICD-9 Codes** | **ICD-10 Codes** |
| --- | --- | --- |
| Myocardial Infarction | 410 - 410.92; 412 | I21 - I22.9; I25.2 |
| Congestive Heart Disease | 428 - 428.9 | I50 - I50.999 |
| Peripheral vascular disease | 440.20 - 440.24; 440.31 - 440.32; 440.8; 440.9; 443.9; 441 - 441.9; 785.4; V43.4 | I70 - I71.9; I73.01; I73.1; I73.9; I79.0; I96; Z95.8 - Z95.9 |
| Cerebrovascular disease | 430 - 438.9 | G45 - G46.999; I60 - I69.999 |
| Dementia | 290 - 290.9 | F00 - F03.999; F05 - F05.999 |
| Chronic pulmonary disease | 490 – 496; 500 – 505; 506.4 | J40 - J47.999; J60 - J67.999; J68.4 |
| Rheumatologic disease | 710.0; 710.1; 710.4; 714.0 - 714.2; 714.81; 725 | M05 - M06.999; M32 - M34.999; M35.3 |
| Peptic ulcer disease | 531 - 534.91 | K25 - K28.999; K56.60 |
| Mild liver disease | 571.2; 571.5; 571.6; 571.4 - 571.49 | K70.0 - K70.31; K73 - K74.999; K75.4 |
| Moderate or Severe Liver  Disease | 572.2 - 572.8; 456.0 - 456.21 | I85.00 - I85.01; I85.10 - I85.11; K70.41; K71.11 - K72.01; K72.10 -K72.11; K72.90 - K72.91; K76.6 - K76.7 |
| Diabetes | 250 - 250.33; 250.7 - 250.73 | E10.10 - E10.11; E10.51 - E10.52; E10.59; E10.641; E10.65; E10.69; E10.9; E11.00 - E11.01; E11.51 - E11.52; E11.59; E11.641; E11.65; E11.69; E11.9; E13.00 - E13.01; E13.10 - E13.11; E13.51 - E13.52; E13.59; E13.641; E13.9 |
| Diabetes with chronic complications | 250.4 - 250.63 | E10.2 - E10.5; E10.61 - E10.619; E11.2 - E11.5; E11.61 - E11.619; E13.2 - E13.5; E13.61 - E13.619 |
| Hemiplegia or paraplegia | 344.1; 342 - 342.92 | G04.1; G81 - G82.999 |
| Renal disease | 582 - 582.9; 583 - 583.7; 585 – 586; 588 - 588.9 | N03.0 - N03.9; N05.2 - N05.5; N05.9; N06.2 - N06.5; N07.2 -N07.5; N08; N17.1 - N17.2; N18.1 - N18.6; N18.9; N19; N25.0; N25.1; N25.81; N25.89; N25.9 |
| Malignancy, including leukemia and lymphoma | 140 - 172.9; 174 - 195.8; 200 - 208.91 | C00 - C26.999; C30 - C34.999; C37 - C41.999; C43 - C43.999; C45 - C45.7; C46 - C58.999; C60 - C76.999; C81 - C85.999; C86 - C86.999; C88 - C88.999; C90 - C97.999; D03.0; D03.10 - D03.12; D03.20 - D03.22; D03.30; D03.39; D03.4; D03.51 - D03.52; D03.59; D03.60 - D03.62; D03.70 - D03.72; D03.8; D03.9; D45 |
| Metastatic solid tumor | 196 - 199.1 | C45.9; C77 - C80.999 |
| AIDS | 042 - 044.9 | B20 - B20.999 |
