# Supplementary material for: Current and historic patterns of chronic disease burden are associated with physical activity and sedentary behavior in older adults: an observational study
Source: BMC Public Health. 2025 Mar 17;25:1032. doi: 10.1186/s12889-025-22264-8 (PMC11917095; doi:10.1186/s12889-025-22264-8)
Supplement: Supplementary file 2 — Supplementary Material 2 [file 12889_2025_22264_MOESM2_ESM.docx]

**Supplemental File 2**. Participant Flow diagram for ACT -AM participants’ inclusion in the presented analyses
