# Supplementary material for: Current and historic patterns of chronic disease burden are associated with physical activity and sedentary behavior in older adults: an observational study
Source: BMC Public Health. 2025 Mar 17;25:1032. doi: 10.1186/s12889-025-22264-8 (PMC11917095; doi:10.1186/s12889-025-22264-8)
Supplement: Supplementary file 3 — Supplementary Material 3 [file 12889_2025_22264_MOESM3_ESM.docx]

**Supplemental File 3.** Sedentary behavior and physical activity summaries and estimated associations with current chronic disease burden (CCI_current_) from models that additionally adjust for smoking, alcohol use, and marital status

|  |  | Overall  N=882 | CCI_current_ = 0  N=513 | CCI_current_ = 1  N=136 | CCI_current_ = 2+  N=233 | Cross-Sectional Modeling Results for CCI_current_  β (95% CI)^d^ |
| --- | --- | --- | --- | --- | --- | --- |
| Sedentary Behavior Measures | **Daily Total Sitting (minutes)^a^, mean (SD)** | 599 (116) | 583 (114) | 598 (111) | 633 (117) | 5.1 (-0.7, 10.8) |
|  | **Mean Bout Duration (minutes)^a^, mean (SD)** | 15.6 (7.3) | 14.7 (6.4) | 15.0 (5.2) | 18.0 (9.4) | 0.5 (0.03, 1.0)* |
|  | **Sitting bouts >30 mins (n/day)^a^, mean (SD)** | 5.8 (1.7) | 5.6 (1.7) | 5.8 (1.7) | 6.2 (1.7) | 0.0 (-0.03, 0.1) |
|  | **Sit-to-stand transitions (n/day)^a^, mean (SD)** | 43.7 (12.8) | 44.8 (12.9) | 43.9 (11.9) | 41.2 (12.9) | -0.6 (-1.2, -0.02)* |
| Light-Intensity Movement Measures | **Standing Time (minutes)^a^, mean (SD)** | 243 (95) | 253 (91) | 238 (88) | 225 (104) | -1.7 (-6.8, 3.4) |
|  | **LPA (minutes)^b,c^, mean (SD)** | 277 (76) | 288 (75) | 267 (67) | 258 (78) | -1.7 (-5.1, 1.6) |
| Moderate-to-Vigorous Intensity Movement Measures | **Steps^a^, mean (SD)** | 6898 (3510) | 7658 (3590) | 6621 (2890) | 5387 (3143) | -304 (-417, -192)* |
|  | **MVPA (minutes)^b,c^, mean (SD)** | 69 (44) | 78 (44) | 68 (39) | 49 (39) | -3.7 (-5.1, -2.4)* |

^a^ activPAL measures: daily total sitting time, mean sitting bout duration, number daily sitting bouts >30 mins, number daily sit-to-stand transitions, daily total standing time, daily total steps

^b^ ActiGraph measures: daily total LPA, daily total MVPA

^c^ LPA and MVPA defined using Objective Physical Activity and Cardiovascular Health in older Women (OPACH) cutpoints, which are validated for an older adult population

^d^ The β parameter (with 95% CI) corresponds to the estimated change in the mean of the given sedentary behavior or physical activity measure associated with a 1-unit increase in CCI_current_; model adjusted for age, sex, race/ethnicity, education, BMI, depressive symptoms, smoking, alcohol, marital status, and device wear time

Notes: CCI = Charlson Comorbidity Index; CI = Confidence Interval; PA = Physical Activity; LPA = Light-Intensity Physical Activity; MVPA = Moderate-to-Vigorous Physical Activity

*Statistically significant associations at the p<0.05 level
