# Supplementary material for: Current and historic patterns of chronic disease burden are associated with physical activity and sedentary behavior in older adults: an observational study
Source: BMC Public Health. 2025 Mar 17;25:1032. doi: 10.1186/s12889-025-22264-8 (PMC11917095; doi:10.1186/s12889-025-22264-8)
Supplement: Supplementary file 4 — Supplementary Material 4 [file 12889_2025_22264_MOESM4_ESM.docx]

**Supplemental File 4.** Sensitivity analyses^e^ of sedentary behavior and physical activity associations with current chronic disease burden (CCI_current_) from models that additionally adjust for smoking, alcohol use, and marital status

|  |  | CCI_current_ = 0  N=513 | CCI_current_ = 1  N=136  β_1vs.0_ (95% CI) | CCI_current_ = 2  N=114  β_2vs.0_ (95% CI) | CCI_current_ = 3+  N=119  β_3+vs.0_ (95% CI) | P-value^d^ |
| --- | --- | --- | --- | --- | --- | --- |
| Sedentary Behavior Measures | **Daily Total Sitting (minutes)^a^** | Reference | 5.0 (-17.2, 27.2) | 18.6 (-7.6, 44.7) | 33.6 (7.5, 59.6) | 0.072 |
|  | **Mean Bout Duration (minutes)^a^** | Reference | -0.6 (-1.6, 0.5) | 2.3 (0.3, 4.3) | 2.3 (0.2, 4.4) | 0.004* |
|  | **Sitting bouts >30 mins (n/day)^a^** | Reference | 0.0 (-0.3, 0.3) | 0.1 (-0.3, 0.4) | 0.3 (0.0, 0.6) | 0.403 |
|  | **Sit-to-stand transitions (n/day)^a^** | Reference | 0.6 (-1.7, 2.9) | -1.6 (-4.4, 1.1) | -2.6 (-5.4, 0.2) | 0.149 |
| Light-Intensity Movement Measures | **Standing Time (minutes)^a^** | Reference | -0.1 (-19.8, 19.5) | -10.2 (-33.8, 13.4) | -17.2 (-40.1, 5.7) | 0.455 |
|  | **LPA (minutes)^b,c^** | Reference | -8.5 (-20.5, 3.5) | -17.2 (-31.5, -2.9) | -12.0 (-27.0, 3.1) | 0.075 |
| Moderate-to-Vigorous Intensity Movement Measures | **Steps^a^** | Reference | -427 (-906, 51) | -808 (-1409, -206) | -1437 (-2008, -867) | <0.001* |
|  | **MVPA (minutes)^b,c^** | Reference | -3.1 (-9.4, 3.2) | -8.8 (-15.9, -1.7) | -18.6 (-25.1, -12.0) | <0.001* |

^a^ activPAL measures: daily total sitting time, mean sitting bout duration, number daily sitting bouts >30 mins, number daily sit-to-stand transitions, daily total standing time, daily total steps

^b^ ActiGraph measures: daily total LPA, daily total MVPA

^c^ LPA and MVPA defined using Objective Physical Activity and Cardiovascular Health in older Women (OPACH) cutpoints, which are validated for an older adult population

^d^ P-value corresponds to a joint (omnibus) test of the 3 parameters corresponding to the exposure contrasts (β_1vs.0_, β_2vs.0_, β_3+vs.0_)

^e^ Sensitivity analyses in which CCI_current_ is modeled as a categorical variable (0, 1, 2, 3+) rather than as a continuous term; model adjusted for age, sex, race/ethnicity, education, BMI, depressive symptoms, smoking, alcohol, marital status, and device wear time

Notes: CCI = Charlson Comorbidity Index; CI = Confidence Interval; PA = Physical Activity; LPA = Light-Intensity Physical Activity; MVPA = Moderate-to-Vigorous Physical Activity

*Statistically significant associations at the p<0.05 level
