# Supplementary material for: Current and historic patterns of chronic disease burden are associated with physical activity and sedentary behavior in older adults: an observational study
Source: BMC Public Health. 2025 Mar 17;25:1032. doi: 10.1186/s12889-025-22264-8 (PMC11917095; doi:10.1186/s12889-025-22264-8)
Supplement: Supplementary file 5 — Supplementary Material 5 [file 12889_2025_22264_MOESM5_ESM.docx]

**Supplemental File 5.** Comparison of sedentary behavior and physical activity between participants^e^ with distal vs. recent multimorbidity onset from models that additionally adjust for smoking, alcohol use, and marital status

|  | Recent onset  (<8 years)  N=105 | Distal onset  (≥8 years)  N=118 | Adjusted difference in means  Β_Distal-Recent_ (95% CI)^d^ |
| --- | --- | --- | --- |
| Mean Bout Duration (min)^a^, mean (SD) | 16.6 (6.3) | 19.3 (11.1) | 2.6 (0.1, 5.1)* |
| Steps^a^, mean (SD) | 6025 (3351) | 4601 (2516) | -1148 (-1859, -437)* |
| MVPA (min)^b,c^, mean (SD) | 58 (41) | 39 (32) | -12.2 (-21.1, -3.4)* |

^a^ activPAL measures: mean sitting bout duration, daily total steps

^b^ ActiGraph measure: daily total MVPA

^c^ MVPA defined using Objective Physical Activity and Cardiovascular Health in older Women (OPACH) cutpoints, which are validated for an older adult population

^d^ The β_Distal-Recent_ parameter (with 95% CI) corresponds to the estimated model-adjusted difference in means of the given activity measure between participants with distal multimorbidity onset vs. recent multimorbidity onset; model adjusted for age, sex, race/ethnicity, education, BMI, depressive symptoms, and device wear time

^e^ Among participants with current chronic disease multimorbidity (CCI_current_ = 2+) and at least 10 years of historical data

Notes: CI = Confidence Interval; PA = Physical Activity; MVPA = Moderate-to-Vigorous Physical Activity

*Statistically significant associations at the p<0.05 level
